# Low adherence to aspirin and calcium carbonate for preeclampsia prevention in pregnant women with chronic hypertension in a brazilian hospital

**DOI:** 10.61622/rbgo/2025rbgo77

**Published:** 2025-11-18

**Authors:** Bruno Verri Jardine, Priscila Oliveira Barbosa, Luiz Sérgio Lima-Júnior, Ranieri Andrade Alves, Taís Delazari de Carvalho, Samuel Luiz Nunes Santos, Ricardo Carvalho Cavalli

**Affiliations:** 1 Universidade de São Paulo Ribeirão Preto Medical School Department of Gynecology and Obstetrics Ribeirão Preto SP Brazil Department of Gynecology and Obstetrics, Ribeirão Preto Medical School, Universidade de São Paulo, Ribeirão Preto, SP, Brazil.

**Keywords:** Aspirin, Calcium carbonate, Hypertension, Pre-eclampsia, Blood pressure, Incidence, Pregnant people, Surveys and questionnaires

## Abstract

**Objective:**

To evaluate adherence to preeclampsia prophylaxis with aspirin and calcium carbonate among pregnant women with chronic hypertension attending a specialized hypertension in pregnancy center at a tertiary hospital in Brazil.

**Methods:**

A cross-sectional study was conducted at the Hospital das Clínicas de Ribeirão Preto. Adherence to aspirin and calcium carbonate was assessed using the Morisky-Green questionnaire. Additionally, we assessed the knowledge of these women regarding preeclampsia. A total of 101 pregnant women were interviewed, and 98 were included in the final analysis.

**Results:**

Non-adherence rates were 64.3% for aspirin and 59.2% for calcium carbonate. The overall incidence of preeclampsia was 22.4% with 11% requiring magnesium sulphate for blood pressure control. No significant differences in preeclampsia incidence were observed among adherent and non-adherent groups for either aspirin or calcium carbonate. Most participants demonstrated high (78.6%) or medium (18.4%) levels of knowledge about preeclampsia.

**Conclusion:**

There was low adherence to aspirin and calcium carbonate prophylaxis among pregnant women with chronic hypertension, with no difference in preeclampsia prevention between adherent and non-adherent groups. These findings raise important questions about the effectiveness of aspirin in reducing preeclampsia risk in chronic hypertension. Further studies should be conducted to evaluate the reasons for low adherence.

## Introduction

Hypertensive disorders of pregnancy (HDP) affect millions of pregnancies around the world and remain a significant public health challenge, particularly in low- and middle-income countries.^(1)^ Among these, preeclampsia – a condition characterized by elevated blood pressure accompanied by proteinuria and/or damage to target organs – represents an important disorder thar contribute to maternal and perinatal morbidity and mortality.^(2)^ In Latin America and the Caribbean, the prevalence of preeclampsia is estimated to be around 6.6%, with even higher rates reported in Brazil, where it affects approximately 9.1% of pregnancies.^(3)^

Preeclampsia is often the most severe HDP, posing both mother and fetus at risk. Pharmacological treatment to reduce the occurrence of preeclampsia is standardized, with low dose aspirin (50-150 mg) established as the primary preventive therapy.^(4)^ In some populations, particularly those with insufficient dietary calcium intake, calcium supplementation is also recommended to further mitigate risks in high-risk pregnancies.^(4)^ In Brazil, the standard recommendation for high-risk women is 100 mg of aspirin daily taken at night, provided through the public healthcare system.^(5)^ Moreover, calcium carbonate supplementation (1-2 g/day) is routinely prescribed for pregnant women with chronic hypertension,^(5)^ and recent national guidelines have expanded this recommendation to include all pregnant women, regardless of risk status.^(6)^ Public health initiatives in Brazil, such as those described by Braga et al. (2024) have emphasized the importance of calcium and aspirin supplementation to reduce maternal morbidity and mortality.^(7)^

While prophylaxis offers a straightforward and effective approach to lowering the risk of preeclampsia, adherence to the prescribed regimen plays a crucial role in its success. Studies indicate that adherence rates exceeding 90% provide the strongest protective effect.^(8,9)^ However, most of the research evaluating the effectiveness of preeclampsia prophylaxis has been conducted in controlled clinical trial settings, predominantly in high-income countries, where adherence is typically higher.

In contrast, real-world data, especially from low- and middle-income countries, remains scarce, even though these regions experiencing a high incidence of preeclampsia. Poor adherence in such regions may further diminish the effectiveness of the prophylaxis treatment. Therefore, to address this gap, we conducted this study to evaluate the adherence of pregnant women with chronic hypertension to aspirin and calcium carbonate as preeclampsia prophylaxis.

## Methods

A cross-sectional study was conducted among pregnant women with chronic hypertension receiving care at the High-Risk Outpatient Clinic of the Ribeirão Preto Medical School Hospital, University of São Paulo. Data were collected using three questionnaires: two based on the Morisky-Green test (to assess adherence to aspirin and calcium carbonate, respectively), and a third developed to assess patient's understanding of chronic hypertension and its potential maternal and fetal consequences.

Pregnant women aged 18 years or older, with a gestational age of more than 20 weeks, and a diagnosis of chronic hypertension – either self-reported or diagnosed before 20 weeks of gestation. All participants were receiving aspirin and calcium carbonate for preeclampsia prophylaxis.

Incomplete or unreliable questionnaire data, inconclusive diagnoses, multiple pregnancies, fetal malformations, and failure to initiate prophylaxis. Patients whose delivery did not occur at the study center were excluded from outcome analyses but no from adherence and knowledge assessment.

The Morisky-Green questionnaire was applied to assess adherence to aspirin and calcium carbonate.^(10)^ The test comprises four Yes/No questions, with each "Yes" scored as 1 point. A total score of 0-1 indicated adherence, while a score of 2-4 indicated non-adherence.^(11)^ An additional question asked whether the participant forgot to take the medication during the previous week. Patients who reported missing medication for more than 15% of the week were considered non-adherent, regardless of their score on the first four questions.

Knowledge of preeclampsia was assessed using a four-question survey adapted from a previously validated instrument and used by other authors.^(12,13)^ The questions addressed the definition, symptoms, complications (maternal and fetal), and source of information. Scores were categorized as follows: 0-1 correct answers = low knowledge; 2 = medium knowledge; 3–4 = high knowledge.

On the day the questionnaire was administered, we recorded systolic and diastolic blood pressure measured by the team of the hospital.

- Maternal background: Data obtained from the medical records included skin color, pregnancy planning status (planned or unplanned), and, if unplanned, the contraceptive method used. We also documented whether the patient was an active or passive smoker and documented alcohol and drug use.
- Clinical indicators: Additional data included the need for blood pressure control using magnesium sulfate, the development of preeclampsia, the patient's age at diagnosis, and the reason for diagnosis (e.g., proteinuria, systemic compromise, imminent eclampsia).
- Delivery outcomes: We recorded the type of delivery, gestational age at delivery, birth weight, and Apgar scores at 1 and 5 minutes.

Data were presented as mean ± standard deviation or as frequencies (%). Group comparisons between adherent and non-adherent patients were conducted using t-tests for continuous variables and chi-square tests for categorical variables. A significance level of p < 0.05 was adopted. All analyses were conducted using SAS 9.4 software. Multivariate analysis was not performed.

This study was evaluated and approved by the Department of Gynecology and Obstetrics and by the Ethical Research Committee of the Ribeirão Preto Medical School Hospital, University of São Paulo (approval no 5.918.852) Certificado de Apresentação de Apreciação Ética: 64491422.0.0000.5440.

## Results

Initially, we interviewed 101 pregnant women who were using aspirin and calcium carbonate for preeclampsia prophylaxis. Three women were excluded due to multiple pregnancies. For 10 participants, delivery data were unavailable because they gave birth at hospitals outside our data access network; however, they were retained in the study for other analyses. Among the 98 pregnant women included in the final analysis, 22 (22.4%) developed preeclampsia, and 11 (11.2%) required magnesium sulfate for blood pressure control. The characteristics of these participants are summarized in table 1.

**Table 1:** Characteristics of the 99 women included in this study

		Average ± SD or Frequencies n(%)
Age (Years)		32.97 ± 5.75
Systolic blood pressure (mmHg)		131.96 ± 15.41
Diastolic blood pressure (mmHg)		81.29 ± 12.81
Skin color		
	Black	20(20.4)
	Brown	24(24.5)
	White	54(55.1)
Parity		
	Nuliparity	17(17.5)
	Multiparity	80(82.5)
Active smoking		
	Yes	11(11.3)
	No	86(88.7)
Passive smoking		
	Yes	17(17.5)
	No	80(82.5)

SD - standard deviation

In relation to adherence to aspirin for preeclampsia prophylaxis, 35 (35.7%) pregnant women demonstrated good adherence, while the majority, 63 (64.3%), had poor adherence. We evaluated the characteristics and obstetric outcomes according to adherence levels (Table 2), but no statistically significant differences were observed between adherent and non-adherent groups.

**Table 2:** Adherence of pregnant women with chronic hypertension to aspirin prophylaxis

Variables		Adherent (n=35)*	Non-Adherent (n=63)*	*p-value*
Skin color				
	Black	7(20)	13(20.6)	0.1798
	Brown	5(14.3)	19(30.2)	
	White	23(65.7)	31(49.2)	
Systolic blood pressure (mmHg)		131.2 ± 14.65	132.34 ± 15.9	0.7431
Diastolic blood pressure (mmHg)		82.03 ± 13.19	80.9 ± 12.69	0.6852
Preeclampsia onset				
	Yes	8 24.2)	15(26.3)	0.8280
	No	25(75.8)	42(73.7)	
Magnesium sulphate				
	Yes	4(12.1)	8(14)	0.7969
	No	29(87.9)	49(86)	
Age at delivery (weeks)		37.5 ± 1.95	37.3 ± 1.4	0.5953
Birth weight				
	Low (< 2,500 g)	6(19.3)	10(18.2)	0.8932
	Normal (> 2,501 g)	25(80.7)	45(81.8)	

* The sample size (n) is smaller for obstetric data due to missing delivery information for some participants

A similar pattern was observed for calcium carbonate: 40.8% of the women had good adherence, while 59.2% had poor adherence. As with aspirin, no statistically significant differences were found in the comparison of clinical and obstetric variables between adherent and non-adherent participants (Table 3).

**Table 3:** Adherence of pregnant women with chronic hypertension to calcium carbonate prophylaxis

Variables		Adherent (n=40)*	Non-Adherent (n=58)*	p-value
Skin color				
	Black	6(14)	14(24.1)	0.2558
	Brown	8(20)	16(27.6)	
	White	26(65)	28(48.3)	
Systolic blood pressure (mmHg)		132 ± 14.95	131.93 ± 15.83	0.9828
Diastolic blood pressure (mmHg)		81.47 ± 10.82	81.18 ± 14.07	0.9075
Preeclampsia onset				
	Yes	11(29.7)	12(22.6)	0.4481
	No	26(70.3)	41(77.4)	
Magnesium sulfate				
	Yes	6(16.2)	6(11.3)	0.5014
	No	31(83.8)	47(88.7)	
Age at delivery (weeks)		37.2 ± 2.03	37.54 ± 1.26	0.3695
Birth weight				
	Low (< 2,500g)	8(22.9)	8(15.7)	0.4012
	Normal (> 2,501 g)	2(77.1)	43(84.3)	

* The sample size (n) is smaller for obstetric data due to missing delivery information for some participants.

Regarding the knowledge of preeclampsia, most pregnant women demonstrated high knowledge about preeclampsia: 77 (78.6%) had high knowledge, 18 (18.4%) had medium knowledge, and only 3 (3%) had low knowledge. When asked about their source of information, 57 (58.2%) reported receiving guidance from physicians, 31 (31.6%) cited the internet, 7 (7.1%) mentioned family members, and 3 (3%) referred to other sources. Complete responses are presented in table 4.

**Table 4:** Knowledge about preeclampsia by pregnant women with chronic hypertension

Variables		n(%) n= 98
What is preeclampsia?		
	Correct	91(92.9)
	Incorrect	7(7.1)
Signs and symptons of preeclampsia		
	Correct	85(86.7)
	Incorrect	13(13.3)
Maternal complications of preeclampsia		
	Correct	64(65.3)
	Incorrect	34(34.7)
Fetal complications of preeclampsia		
	Correct	75(76.5)
	Incorrect	23(23.5)
Total score of preeclampsia knowledge		
	High	77(78.6)
	Medium	18(18.4)
	Low	3(3)
Where did you obtain the information?		
	Family	7(7.1)
	Internet	31(31.6)
	Physician	57(58.2)
Others		3(3)

## Discussion

The objective of our study was to evaluate adherence to preeclampsia prophylaxis in women with chronic hypertension attending a high-risk hospital in Brazil. Our findings raise important questions and highlight the need for further studies on the use of preeclampsia prophylaxis: 1) the majority of pregnant women were non-adherent to prophylactic treatment, with 64.3% not adhering to aspirin and 59.2% to calcium carbonate, emphasizing the need for targeted educational initiatives to stress the importance of adherence in high-risk pregnancies. 2) the overall incidence of preeclampsia was 22.4%, with a similarly high incidence observed in both adherent and non-adherent groups for aspirin and calcium carbonate, suggesting that these interventions may be less effective in women with chronic hypertension. 3) most women demonstrated medium to high knowledge about preeclampsia, with physicians as the main source of information, followed by the internet. These results highlight the crucial role of healthcare professionals in patient education and the importance of providing reliable, accessible online resources to empower women with accurate information.

Adherence is crucial for successful treatment; however, low adherence to prescribed medications is a common issue influenced by various factors.^(14)^ In our study, only 35.7% of women reported good adherence to aspirin. Interestingly, these results are comparable to those from high-income countries. For example, a study by Abheiden et al.^(15)^ in Amsterdam reported a non-adherence rate of 46.3%, while another study in Australia found a non-adherence rate of 44%.^(16)^ These similarities highlight the widespread challenge of ensuring adherence to preeclampsia prophylaxis, even in countries with greater resources. It is important to note that our data, along with the findings from Amsterdam and Australia, reflect real-world adherence, which often differs from clinical trials where adherence rates typically exceed the 80% duet to close monitoring.^(9,17,18)^ Unfortunately, data on adherence to preeclampsia prophylaxis from low- and middle-income countries remain scarce,^(19)^ reinforcing the need for more research focused on adherence among pregnant women at risk in these regions. We believe that disparities between countries — such as differences in prescription access, education levels, and healthcare infrastructure — may further influence adherence to preeclampsia prophylaxis.

Low-dose aspirin is widely recognized as a first-line medication for preeclampsia prevention, with strong evidence supporting its effectiveness. When started at the end of the first trimester and continued until 36 weeks of gestation, aspirin can reduce the risk of preeclampsia by approximately 18% and is also associated with lower rates of small-for-gestational-age births and preterm delivery.^(20)^ However, in our study, the incidence of preeclampsia was 22.4%, with no significant difference between adherent (24.2%) and non-adherent (26.3%) women. This suggests that, in this population, adherence to aspirin did not reduce the risk of development of preeclampsia. Our findings are consistent with previous studies that have reported limited effectiveness of aspirin in preventing preeclampsia among women with chronic hypertension.^(21–23)^ Randomized clinical trials comparing aspirin with placebo in high-risk pregnant women have also failed to show a significant benefit when chronic hypertension was the primary risk factor.^(22,23)^ These results raise the possibility that aspirin may not be as effective in reducing preeclampsia risk among women with chronic hypertension, the condition shared by all participants in our study.

Calcium supplementation is another recommended strategy for the prevention of hypertensive disorders during pregnancy, particularly in women at higher risk of preeclampsia or with low dietary calcium intake.^(24)^ The World Health Organization (WHO) recommends 1.5-2 g of calcium, divided into three or more doses, taken with meals and separately from iron supplements.^(25)^ Although aspirin remains the first-line prophylactic treatment, calcium supplementation has received increasing attention. Recently, the Brazilian Ministry of Health issued new guidelines recommending calcium supplementation for all pregnant women, regardless of individual risk, as part of routine prenatal care.^(6)^

Despite this, adherence to calcium supplementation has been less studied compared to aspirin. A study conducted in Ethiopia evaluated adherence patterns and found no significant differences between intake regimens (e.g., doses taken with or without iron), with adherence rates above 75%.^(26)^ However, that study included additional counseling and provided calendar reminders, which likely contributed to the high adherence rates. As with aspirin, these findings underscore that adherence tends to be higher when actively monitored or supported—common in clinical trial settings. This highlights the critical role of healthcare providers in reinforcing the importance of calcium supplementation through clear guidance, education, and ongoing support during prenatal care.

As education and understanding of health conditions are critical factors for adherence to prescribed treatments,^(27,28)^ we hypothesized that pregnant women's knowledge of preeclampsia would be associated with their adherence to prophylaxis. Most participants demonstrated high or medium levels of knowledge about preeclampsia, with physicians identified as the primary source of information. These findings are consistent with previous studies showing that healthcare professionals play a key role in promoting adherence to preventive therapies.^(28,29)^ However, the significant role of the internet as a secondary source underscores the need for accessible, reliable, and evidence-based online materials to support and reinforce the education provided during clinical care.

Our study is the first to report adherence to preeclampsia prophylaxis among pregnant women with chronic hypertension in Brazil. While our findings provide valuable insight into real-world adherence rates in a low- and middle-income country setting, and include both aspirin and calcium carbonate prophylaxis, there are limitations that must be considered. The single-center design and reliance on self-reported adherence may introduce bias and limit the generalizability of our results, particularly given the complexity of cases managed at our hospital. Additionally, the lack of a significant association between adherence and preeclampsia incidence highlights the need for further investigation in larger, multicenter studies.

We believe our results raise important questions and underscore the need for more research into the effectiveness of aspirin in different high-risk populations. Despite global recommendations to initiate aspirin in pregnant women at increased risk for preeclampsia, protocols are not standardized across countries. In some settings, aspirin is recommended before 16 weeks of gestation and no later than 20 weeks, whereas in others, initiation is accepted up to 22 weeks. Furthermore, the definition of low-dose aspirin varies widely, ranging from 50 to 150 mg, often depending on the formulations available within each healthcare system.

## Conclusion

Our findings highlight low adherence to preeclampsia prophylaxis among pregnant women with chronic hypertension. Notably, the incidence of preeclampsia did not differ significantly between adherent and non-adherent participants. These results underscore the need for further studies to better understand the barriers to adherence and to assess the effectiveness of aspirin prophylaxis in this specific high-risk population. Improving access to reliable information, both through healthcare providers and digital platforms, is essential to reducing preeclampsia-related complications.
